# University of Maryland, Etc.

**Published:** 1880-03

**Authors:** 


University of Maryland.—We have incidentally learned that this old school contemplates adopting the graded three years' term of lectures, before permitting its students to apply for final examination for the degree of M. D. We are glad of this, and would be pleased to learn that all the schools, or such of them, at least, as are deserving of confidence and patronage, had decided on a similar course. The two oldest schools in our country, and justly distinguished throughout their career, viz.: the University of Pennsylvania and Harvard University, have been successful in their "new departure" thus far, and we feel confident will continue to receive the patronage their high standard of admission to the doctorate so justly entitles them; and it may be confidently asserted of their graduates, that they are the peers of those from any medical schools wherever located. How grandly, and proudly, too, such institutions stand out on the theatre of professional life, and dwarf into utter insignificance the school that attempts to draw large numbers of verdant, unsuspecting young and old men, too, to its lecture-rooms by flooding the country with blank invitation certificates, to be filled by the party receiving them; and from one to two more, to be handed to a friend or friends engaged in studying medicine, for their endorsement, in like manner, so as to secure their connection with such school! We thought that some of the medical schools had opened their doors quite wide enough, a few years ago, when they offered to receive students on the endorsement of congressmen, legislators, etc.; but we were totally unprepared to comprehend the depths of depravity to which a school might descend, when guilty of such conduct, as we have stated in connection with these certificates. It has been stated, upon what is believed to be reliable authority, that certain districts of Pennsylvania have been strewn broadcast with such certificates, and that these reprehensible documents have also been sent into most of the States and Territories with a liberal hand. Now, a school that has not sufficient talent to attract students to its lecture-rooms, without resorting to the contemptible procedure under consideration, should be abolished immediately by the strong arm of the law, as no amount of public reprobation, however urgently applied, would be likely to arrest it in its course of injury to the profession and the community at large. The reputed conduct of such a faculty would seem to give some significance to the remark of a certain "professor," a short while ago, that whether a man would make a doctor or a donkey of himself would depend upon the money he might have. We repeat again, we are glad the University of Maryland contemplates making the change to a three years' course of study. Anything that looks toward elevating the profession is hailed with delight by us in these degenerate times.

